# Two Cases of the *MYH9* Disorder Fechtner Syndrome Diagnosed from Observation of Peripheral Blood Cells before End-Stage Renal Failure

**DOI:** 10.1155/2019/5149762

**Published:** 2019-11-26

**Authors:** Shin Teshirogi, Jun Muratsu, Hidenori Kasahara, Ken Terashima, Sho Miki, Tomohiro Minami, Yujiro Okute, Suguru Yoneda, Atsuyuki Morishima, Shinji Kunishima, Katsuhiko Sakaguchi

**Affiliations:** ^1^Department of Nephrology and Hypertension, Sumitomo Hospital, 5-3-20 Nakanoshima, Kita-ku, Osaka 530-0005, Japan; ^2^Department of Advanced Diagnosis, Clinical Research Center, National Hospital Organization Nagoya Medical Center, Nagoya, Japan

## Abstract

As a *MYH9* disorder, Fechtner syndrome is characterized by nephritis, giant platelets, granulocyte inclusion bodies (Döhle-like bodies), cataract, and sensorineural deafness. Observation of peripheral blood smear for the presence of thrombocytopenia, giant platelets, and granulocyte inclusion bodies (Döhle-like bodies) is highly important for the early diagnosis of *MYH9* disorders. In our two cases, sequencing analysis of the *MYH9* gene indicated mutations in exon 24. Both cases were diagnosed as the *MYH9* disorders Fechtner syndrome before end-stage renal failure on the basis of the observation of peripheral blood smear.

## 1. Introduction

*MYH9* mutations cause the autosomal dominant macrothrombocytopenic syndrome of May-Hegglin anomaly, Fechtner syndrome, Sebastian syndrome, and Epstein syndrome. Recently, these diseases are being referred to as *MYH9* disorders. The MYH9 gene encodes the nonmuscle myosin heavy chain –IIA (NMMHC-IIA). *MYH9* disorders are characterized by giant platelets, thrombocytopenia, and characteristic Döhle-like bodies in granulocytes. Among these diseases, the involvement of Alport syndrome symptoms, namely nephritis, cataract, and sensorineural deafness, is associated with the mutation site of the *MYH9* gene. Fechtner syndrome, a *MYH9* disorder, develops nephritis and end-stage renal failure. In most previous reports of *MYH9* disorders in Japan, Fechtner syndrome was diagnosed after end-stage renal failure. Here, our two cases were diagnosed before end-stage renal failure on the basis of the observation of peripheral blood smear, neutrophil inclusion bodies (Döhle-like bodies), and giant platelets.

In the management of chronic kidney disease, the findings from peripheral blood smear, presence of the Döhle-like bodies in neutrophils, and giant platelets are important for the early diagnosis of *MYH9* disorders, especially if the symptoms are not evident.

## 2. Case Presentation

### 2.1. Case 1

**Figure 1:**
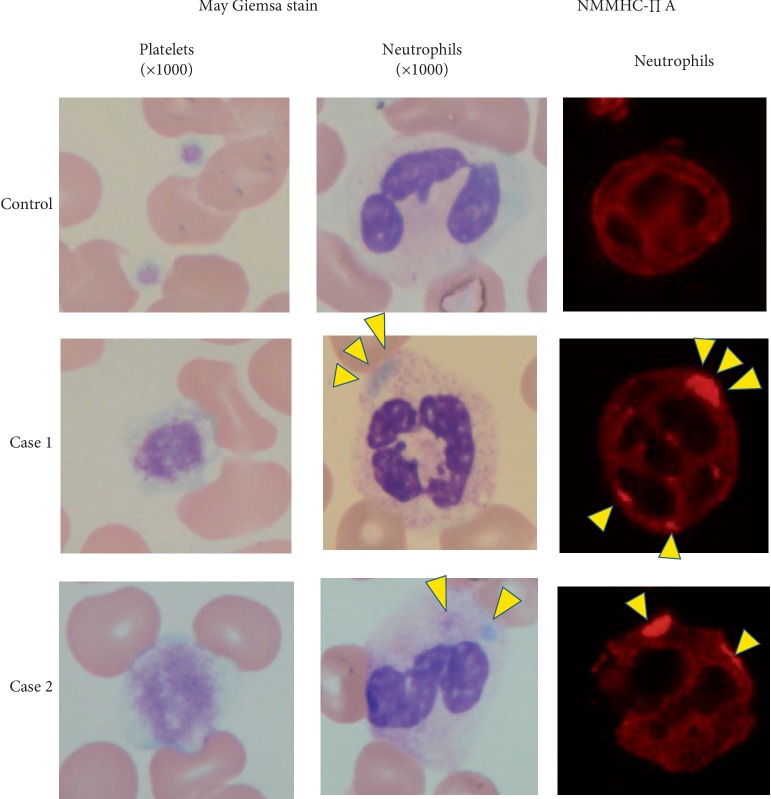
Upper panels present the control samples; middle panels, case 1 samples; and lower panels, case 2 samples. The May-Giemsa-stained platelets (in the left: original magnification ×1000) show giant platelets from the case 1 and 2 samples. In the May-Giemsa-stained neutrophils (in the middle: original magnification ×1000), the cytoplasmic inclusion bodies (Döhle-like bodies) in the case 1 and case 2 samples are indicated with arrowheads. The nonmuscle myosin heavy chain-II A (NMMHC-II A) distribution in neutrophils is shown in the immunofluorescence micrographs of the neutrophils (in the right). NMMHC-II A is diffusely distributed in the control neutrophils. Arrowheads represent the accumulation of granular NMMHC-II A in neutrophils of cases 1 and 2.

**Figure 2:**
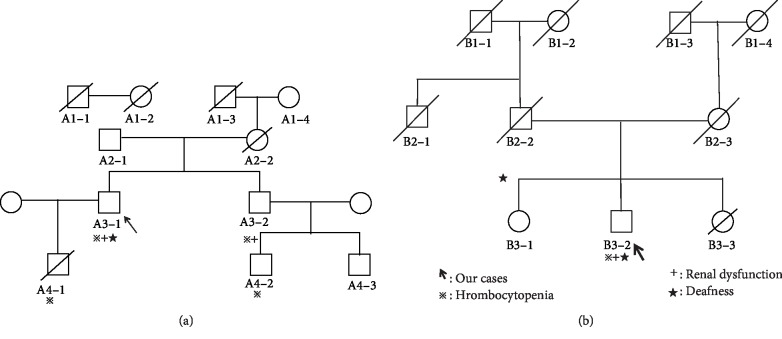
Family pedigree of our cases and sequence electropherogram of the complementary strand of the *MYH9* gene. (a: case 1) Family pedigree of case 1: A1-1 died of tuberculosis at 40 years old. A1-2 died of senility at 90 years old. A1-3 died of gastric cancer at 60 years old. A1-4 had blindness and died of senility at 80 years old. A2-1 had diabetes and was 87 years old. A2-2 died of subarachnoid hemorrhage at 61 years old. A3-1 was case 1. A3-2 was 56 years old. He had thrombocytopenia and renal dysfunction. A4-1 had thrombocytopenia. He died of colon cancer at 29 years old. A4-2 had thrombocytopenia. He was 24 years old. A4-3 had no significant findings. He was 22 years old. (b: case 2) Family pedigree of case 2. B1-1, B1-2, and B1-3 died from senility at around 80 years old. B1-4 died at 60 years old. The cause of her death was unclear. B2-1 died at 62 years old. The cause of his death was unclear. B2-2 had hearing loss. He died of cerebral infarction at 84 years old. B2-3 had hypertension and died from senility at 90 years old. B3-1 was 65 years old. He had no significant findings. B3-2 was case 2. B3-3 had a gallstone. He was 62 years old.

A 56-year-old male was referred to our hospital for the management of chronic kidney disease. At the age of 17 years, hematuria and proteinuria were observed. At the age of 30 years, he presented with thrombocytopenia, sensorineural deafness, and cataract. At the age of 56 years, he was referred to our hospital for the management of hypertension and elevated serum creatinine level. His blood and urinary analysis results are shown in Table 1. In the peripheral blood smear, thrombocytopenia, giant platelets, and neutrophil inclusion bodies (Döhle-like bodies) were observed with May-Giemsa staining (Figure 1). We identified a relevant family history (Figure 2(a)). His son had thrombocytopenia. His mother died of subarachnoid hemorrhage at the age of 61 years, and his younger brother had thrombocytopenia and renal dysfunction.

From these findings, we considered the possibility of *MYH9* disorders and performed immunofluorescence analysis for neutrophil NMMHC-IIA localization [1, 2]. We found a few large NMMHC-IIA aggregates in the neutrophils (Figure 1). Mutational analysis of the *MYH9* gene revealed a heterozygous duplication of 21 nucleotides in exon 24 (p.E1066_A1072dup, c.3195_3215dup; Figure 2(a)).

We started a nutritional therapy and an angiotensin receptor II; antagonist (olemsartan 20 mg/day) to reduce proteinuria.

### 2.2. Case 2

**Figure 3:**
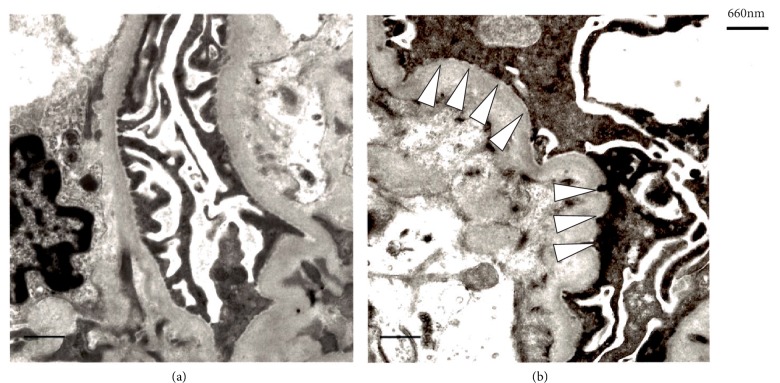
Electron microscopy image of a renal history obtained by percutaneous needle biopsy from case 2 (original magnification ×12000). Normal podocytes and a slit diaphragm are shown (a). In the other field, focal effacement of podocytes and loss of the interpodocyte slit diaphragm are indicated by arrowheads (b).

A 59-year-old male was referred to our hospital because elevated serum creatinine level was indicated in his medical examinations. Renal dysfunction was only recognized at the age of 59 years, when he presented with cataract and sensorineural deafness. In his family history, his father had sensorineural deafness from the age of 20 years and died of cerebral infarction at the age of 84 years. His mother had hypertension from the age of 50 years (Figure 2(b)). Blood and urinary analysis results are shown in Table 1. In the peripheral blood smear, thrombocytopenia, giant platelets, and neutrophil inclusion bodies (Döhle-like bodies) were observed using May-Giemsa staining (Figure 1). We suspected the possibility of a *MYH9* gene abnormality. Immunofluorescence micrographs of neutrophils showed granular accumulation of NMMHC-IIA in the neutrophils (Figure 1). Subsequent genetic mutation analysis revealed p.E1084del mutation (c.3250_3252delGAG) in *MYH9* exon 24 (Figure 2(b)). To determine other factors of renal dysfunction, we performed renal biopsy by percutaneous needle aspiration. In the histological analysis, no significant changes were observed in the mesangium and tubulointerstitial lesions on light microscopy. However, on electron microscopy, focal effacement of podocytes and loss of the interpodocyte slit diaphragm were observed (Figure 3). From these findings, he was diagnosed with Fechtner syndrome, a *MYH9* disorder. Hypertension and additional risk factors of renal failure had been managed mainly with dietary intervention. Even at 5 years after the diagnosis, his creatinine level remained at 1.3 mg/dL.

The institutional review boards of Sumitomo Hospital and Nagoya Medical Center approved this study. Written informed consent was obtained from all the patients in accordance with the principles of the Declaration of Helsinki.

## 3. Discussion

*MYH9* disorders include May-Hegglin anomaly, Sebastian syndrome, Fechtner syndrome, and Epstein syndrome. These are classified according to the presence of giant platelets, granulocyte inclusion bodies (Döhle-like bodies), nephritis, sensorineural deafness, and cataract [1–3] (Table 2). Here, we describe two cases of Fechtner syndrome before end-stage renal failure. In case 1, a large amount of proteinuria was observed. Owing to renal atrophy, we did not perform a renal biopsy. In contrast, in case 2, mild proteinuria was observed. However, electron microscopy revealed focal and segmental effacement of podocytes and loss of the interpodocyte slit diaphragm. The *MYH9* gene encodes the nonmuscle myosin heavy chain –IIA (NMMHC-IIA). NMMHC-II A is an actin-binding protein that also plays an important role in cell adhesion and maintenance of tissue architecture [4]. It forms myosin II A with myosin light chain and is responsible for the contractile mechanism in the foot process of podocytes [5]. In previous studies, these changes were reported to be associated with the loss of NMMHC-II A expression [6]. This falling out of the foot process is responsible for proteinuria [7]. In many previous reports of *MYH9* disorders, cases of Fechtner syndrome complicated with renal failure were diagnosed after end-stage renal failure [8, 9]. Here, our two cases could be diagnosed before end-stage renal failure on the basis of the main findings, including giant platelets and granulocyte inclusion bodies (Döhle-like bodies). As an advantage of diagnosis before end-stage renal failure, erroneous treatment such as immunosuppressive therapy can be prevented. In addition, antihypertensive therapy can be started early, as done in our cases. In previous studies, administration of an angiotensin II receptor blocker or an angiotensin-converting enzyme inhibitor for renal injury associated with *MYH9* disorders was identified to be effective to reduce proteinuria and suppress the development of renal dysfunction [10]. Diagnosis before end-stage renal failure is necessary for the management around the perioperative period, such as hemostasis during maintenance hemodialysis or tube insertion for peritoneal dialysis. Finally, because *MYH9* disorders are genetic diseases, early detection of patients based on family history is also important. We can offer a genetic consultation to patients' families and other relatives. In fact, in the younger brother of case 1, therapeutic intervention started to suppress the development of renal failure. As mentioned earlier, the diagnosis of *MYH9* disorders before end-stage renal failure is meaningful; therefore, careful observation of giant platelets and Döhle-like bodies in patients with chronic kidney disease (CKD) is important. However, in Epstein syndrome, inclusion bodies are difficult to recognize in granulocytes.

Cases with *MYH9* mutations in exon 24 might clearly show Döhle-like bodies and giant platelets. Previously, about 30 mutations of the *MYH9* gene were reported. However, among the 40 exons in the *MYH9* gene, mutations are concentrated in the specific codons of exons 1, 16, 26, 30, 38, and 40 [11]. Both our cases had mutations in exon 24. In previous studies, cases with mutations in exon 24 were very rare [12–14]. Even among the same genetic mutations, the phenotype can vary [15]. Kidney damage occurs in approximately 25% of patients with *MYH9* disorders as progressive proteinuric nephropathy. In most cases, nephropathy occurs before the age of 35 years and presents an aggressive course. In some cases, proteinuria may appear later and/or show a slower progression [16]. Hearing loss occurred in approximately 50% of cases at a mean age of 33 years [17]. As indicated in Table 2, thrombocytopenia is common, but the occurrence of symptoms such as renal disorder, deafness, and cataract differed from each other even in the same genetic mutation. These findings indicate that other factors such as age or sex might be required for the onset and progression of symptoms. Therefore, in cases with thrombocytopenia, attention should be paid to the possibility of *MYH9* disorders.

There still remain many unknown points regarding the pathophysiology of *MYH9* disorders, particularly Fechtner syndrome. Further investigation is required to assess the incidence and mechanism of renal failure in *MYH9* disorders, especially Fechtner syndrome.

In conclusion, in patients with chronic kidney disease, findings of giant platelets and granulocyte inclusion bodies (Döhle-like bodies) are important for the diagnosis of *MYH9* disorders, particularly Fechtner syndrome.

## Figures and Tables

**Table 1:** Clinical characteristics, blood and urinary analysis of these cases.

	Case 1	Case 2	Reference range
Height (cm)	173	164	
Weight (kg)	79.2	64.1	
BMI (kg/m^2^)	26.5	23.8	

*Urinary analysis*			
pH	6.0	5.0	5.0–7.5
Specific gravity	1.003	1.019	1.005–1.030
Protein (g/day)	1.5	0.02	negative
Urine occult blood reaction Cast	1+ fatty cast, epithelial cast and granular cast	Negative hyaline cast, epithelial cast and granular cast	Negative negative

*Blood analysis*			
White blood cell (/*μ*L)	4,300	4,000	3,300–8,600
Red blood cell (×10^6^/*μ*L)	3.00	4.38	4.30–5.60
Hemoglobin (g/dL)	9.8	13.6	13.5–17.0
Hematocrit (%)	29.9	40.9	40.0–51.0
Platelet counts (×10^4^/*μ*L)	8.2	8.7	15.0–35.0
Mean platelet volume (fL)	11.5	13.2	6.8–9.4
Bleeding time (minutes)	1	1	1–5
Sodium (mEq/L)	141	143	143
Potassium (mEq/L)	4.7	4.2	3.6–5.0
Chloride (mEq/L)	109	109	98–108
Calcium (mg/dL)	8.7	8.9	8.2–10.2
Phosphorus (mg/dL)	6.2	2.8	2.7–4.4
Total protein (g/dL)	7.0	6.4	6.7–8.3
Albumin (g/dL)	4.1	4.4	3.8–5.3
Blood urea nitrogen (mg/dL)	78	17	8–20
Creatinine (mg/dL)	5.91	1.24	0.36–1.06
estimate glomerular filtration rate (eGFR) (mL/min/1.73m^2^)	8.8	47.6	
Hemoglobin A1c (NGSP) (%)	5.7	5.3	4.6–6.2
Antinuclear antibody	×40	<×40	<×40

**Table 2:** *MYH9* disorders; May-Hegglin syndrome, Sebastian syndrome, Epstein syndrome and Fechtner syndrome and clinical features. Clinical features of our cases and previous reported cases with the same mutations of the *MYH9* gene in exon 24.

*MYH9* disorders		Giant platelets	Granulocyte inclusion bodies	Nephritis	Sensorineural deafness	Cataract
May-Hegglin syndrome		＋	＋ （large）	－	－	－
Sebastian syndrome		＋	＋ （small）	－	－	－
Epstein syndrome		＋	－	＋	＋	－
Fechtner syndrome		＋	＋	＋	＋	＋

*MYH9* gene mutations	Age and sex	Giant platelets and platelet count (reference range: 15.0-35.0×10^4^/*μ*L)	Granulocyte inclusion bodies	Nephritis	Sensorineural deafness	Cataract

*Case 1*	56	＋	＋	＋	＋	＋
p.E1066_A1072dup	Male	(8.2×10^4^/*μ*)				
N. Pujol Moix et al. [12], De Rocco D et al. [13]	50	＋	＋	－	－	－
p.E1066_A1072dup	Female	(1.7–8.3×10^4^/*μ*)				
De Rocco D et al. [13]	23	＋	＋	－	－	＋
p.E1066_A1072dup	Male	(3.0–8.0×10^4^/*μ*)				
De Rocco D et al. [13]	25	＋	＋	－	－	－
p.E1066_A1072dup	Female	(3.0–8.0×10^4^/*μ*)				

*Case 2*	59	＋	＋	＋	－～±	＋
p.E1084del	Male	(8.7×10^4^/*μ*)				
Miyazaki et al. [14]	21	＋	＋	－	－	－
p.E1084del	Male	(8.5×10^4^/*μ*)				
